# Genetic Characteristics of Porcine Hemagglutinating Encephalomyelitis Coronavirus: Identification of Naturally Occurring Mutations Between 1970 and 2015

**DOI:** 10.3389/fmicb.2022.860851

**Published:** 2022-03-18

**Authors:** Amina Nawal Bahoussi, Yan-Yan Guo, Rui-Zhu Shi, Pei-Hua Wang, Ya-Qian Li, Chang-Xin Wu, Li Xing

**Affiliations:** ^1^Institute of Biomedical Sciences, Shanxi University, Taiyuan, China; ^2^Shanxi Provincial Key Laboratory of Medical Molecular Cell Biology, Shanxi University, Taiyuan, China; ^3^Shanxi Provincial Key Laboratory for Prevention and Treatment of Major Infectious Diseases, Taiyuan, China

**Keywords:** porcine hemagglutinating encephalomyelitis virus, coronavirus, spike glycoprotein, receptor-binding domain, genomic mutation, B cell epitope

## Abstract

Porcine hemagglutinating encephalomyelitis virus (PHEV) is a *Betacoronavirus* characterized by neurological symptoms and a worldwide prevalence. Although PHEV is one of the earliest discovered porcine coronaviruses, it remains poorly studied. The full-length genome of the earliest PHEV strain collected in 1970 in the United States (PHEV/67 N/US/1970) was determined in October 2020. Using this virus as a prototype, we comparatively analyzed all available PHEV full-length sequences during 1970–2015. In phylogenetic trees based on PHEV full-length or spike glycoprotein open reading frame genomic sequences, PHEV/67 N/US/1970 was sorted into a clade different from that of viruses isolated in the United States in 2015. Intriguingly, United States and Belgium viruses isolated in 2015 and 2005, respectively, revealed multiple deletion mutation patterns compared to the strain PHEV/67 N/US/1970, leading to a truncated or a non-functional NS2A coding region. In addition, the genomic similarity analysis showed a hypervariability of the spike glycoprotein coding region, which can affect at least eight potential linear B cell epitopes located in the spike glycoprotein. This report indicates that PHEVs in the United States underwent a significant genetic drift, which might influence PHEV surveillance in other countries.

## Introduction

Coronaviruses (CoVs) rank among the largest groups of enveloped positive-sense, single-stranded RNA viruses, which fall under the *Coronavirinae* subfamily, *Coronaviridae* family, in *Nidovirales* order (Payne, 2017). In humans and vertebrates, the clinical picture of CoVs is dominated by the infection of three major systems, including the respiratory, digestive, and central nervous systems (CNS; Chen et al., 2020). CoVs are classified into four genera: *Alpha-CoV, Beta-CoV, Gamma-CoV*, and *Deltacoronavirus* (de Groot et al., 2012). Human CoVs are generally attributed to a zoonotic origin before leaping into an intermediate host or directly to humans (Guan et al., 2003). The severe acute respiratory syndrome coronavirus 1 (SARS-CoV-1) in 2002/2003 (Ksiazek et al., 2003), the Middle East respiratory syndrome coronavirus (MERS-CoV) in 2012 and the novel severe acute respiratory syndrome coronavirus 2 (SARS-CoV-2) first identified in December 2019 in Wuhan, China (Zhou et al., 2020), confirmed that CoVs are likely originated in animals. The emergence of these three zoonotic severe *Betacoronavirus* outbreaks in less than two decades indicates the adaptation potentials of CoVs, their genetic plasticity, and the expansion of host range, which prompts us to explore the evolutionary characters of another mammal *Betacoronavirus*: the porcine hemagglutinating encephalomyelitis virus (PHEV).

Pigs are a natural host of six viruses from the *Coronaviridae* family (Wang et al., 2019), four of which were identified as *Alpha-CoVs*, including transmissible gastroenteritis virus (TGEV; Doyle and Hutchings, 1946), porcine epidemic diarrhea virus (PEDV; Wood, 1977), porcine respiratory coronavirus (PRCV; Pensaert et al., 1986), and the newly discovered swine acute diarrhea syndrome coronavirus (SADS-CoV; Gong et al., 2017; Pan et al., 2017; Zhou et al., 2018); one *Beta-CoV* PHEV (Greig et al., 1962); and the porcine *Deltacoronavirus* (PDCoV; Woo et al., 2012).

Porcine hemagglutinating encephalomyelitis virus is one of the earliest identified and isolated porcine CoVs and the only known neurotropic virus in pigs (Mora-Diaz et al., 2019). PHEV infection was first reported in the Ontario province of Canada in 1957 (Roe and Alexander, 1958) and the viral pathogen was first isolated from the brains of encephalitis piglets in 1962 (Greig et al., 1962). Since then, PHEV infection has been reported in countries of America, Europe, and Asia, including the United States (Cutlip and Mengeling, 1972), Argentina (Quiroga et al., 2008), Great Britain (Cartwright et al., 1969), Belgium (Sasseville et al., 2001), Japan (Hirano and Ono, 1998), South Korea (Rho et al., 2011), and China (Gao et al., 2011; Dong et al., 2014).

In neonatal pigs, PHEV infection causes vomiting and wasting disease (VWD) or encephalomyelitis (Mengeling et al., 1972; Andries and Pensaert, 1981) with approximately 100% mortality rate and a high prevalence in the United States (Mora-Diaz et al., 2020). The primary replication of PHEV starts in the upper respiratory tract and tonsils, with non-specific clinical signs, including sneezing, coughing, and transient fever of 1–2 days (Andries and Pensaert, 1980; Hirahara et al., 1987). During the incubation, PHEV can penetrate the CNS (Andries and Pensaert, 1980) and invade predominantly neurons with a massive activation of the surrounding microglia and an obvious inflammation of CNS (J. Zhang et al., 2021). During the early stage of PHEV infection, microglial activation leads to a monumental proinflammatory cytokine release (IL-1β, IL-6, TNF-α, and IFN-γ) to limit the viral replication (Zhang et al., 2021). However, an excessive microglial activation at a late stage, with a blood–brain barrier (BBB) destruction and a monocytes/macrophages infiltration of CNS, aggravates PHEV infection (Zhang et al., 2021). Furthermore, PHEV causes lysosomal disorders by decreasing the expression of progranulin protein (Lan et al., 2020), a significant accumulation of autophagosomes (APs) in nerve cells (Li et al., 2021) and other neurological dysfunctions (Mora-Diaz et al., 2021a). Although pigs of any age can be infected, the clinical manifestation depends on the age at PHEV-infection time (Hirano, 2004). In older pigs, PHEV is highly seroprevalent and usually subclinical (Dong et al., 2014; Mora-Diaz et al., 2020). However, a recent report showed the persistent virus shedding in feces and oral fluid with an active humoral immune response (IgM, IgG) in subclinical grower pigs suggesting an active viral replication attenuated by the immune response (Mora-Diaz et al., 2021b).

Porcine hemagglutinating encephalomyelitis virus belongs to *Betacoronavirus* genus*, Embecovirus* subgenus (Zhi et al., 2005; Llanes et al., 2020). The RNA genome of PHEV is about ~30 kb in length and shows a short untranslated region (UTR) at both ends (Fehr and Perlman, 2015). The genome is flanked with a 5′MET cap and a 3′ polyA tail (Fehr and Perlman, 2015) and contains at least 11 predicted open reading frames (ORFs; Vijgen et al., 2006; Shi et al., 2018). The first two-thirds of the viral genome is occupied by replicase ORF which encodes replicase polyprotein, a precursor for multiple nonstructural proteins (Nsps; Sola et al., 2015). The one-third 3′ region of the viral genome encodes five major structural proteins (hemagglutinin-esterase protein, HE; spike glycoprotein, S; envelope protein, E; membrane protein, M; and nucleocapsid protein, N) and three non-structural proteins (NS2A, NS4.9, and NS12.7; Weiss and Navas-Martin, 2005; Vijgen et al., 2006; Wang et al., 2019). Among them, N protein is the most abundant structural protein that binds to viral genome RNA. M and E are membrane proteins (Wang et al., 2019). Whereas, HE protein which is not present in other *Beta-CoVs,* is characterized by hemagglutinating (HA), acetyl-esterase (AE) or receptor-destroying and binding activities (Cornelissen et al., 1997).

The S glycoprotein of coronaviruses is a viral envelope protein with a binding specificity regulated by a receptor-binding domain (RBD; Bosch et al., 2003; Dong et al., 2015). S glycoprotein facilitates the attachment of the virion and its penetration into the host cells by recognizing and interacting with a proper cellular receptor (Schultze et al., 1992). During the entry of viral particles, S glycoprotein is cleaved into two subunits, named S1 and S2 subunits (Walls et al., 2016). S glycoprotein of PHEV is composed of about 1,349 amino acids (aa; Dong et al., 2015) and contains several conserved domains as determined in NCBI conserved domain database (CDD; Marchler-Bauer et al., 2017). The S1 subunit contains N-terminal domain (NTD, aa 16–298) and S1 receptor-binding domain (S1-RBD, aa 311–608), whereas the S2 subunit contains an S2 domain (aa 784–1,276), a transmembrane structure (TM, aa 1,294–1,316), and a cytosolic tail at the C-terminus. The S1-RBD of PHEV has been shown to bind specifically to neural cell adhesion molecules (NCAM), also named CD56 (Dong et al., 2015), which is involved in neuronal infection and injuries (Gao et al., 2010). The S2 subunit controls the membrane fusion between virion and cell during viral entry into the host cells (Bosch et al., 2003; Walls et al., 2016).

In China, PHEV was first reported in a pig farm in Beijing in 1985, then in Jilin, Liaoning, Shandong, and other provinces (Gao et al., 2011; Chen et al., 2012; Dong et al., 2014). In 2007, two pig farms in Changchun City and Siping City, Jilin Province of China, had outbreaks of PHEV, with mortality rates of 47.6 and 100%, respectively (Gao et al., 2011; Shi et al., 2018). In 2015, the influenza-like respiratory disease occurred in display pigs in Michigan, United States, and PHEV was finally identified in the specimens of clinically sick pigs (Lorbach et al., 2017). Currently, there is no commercially available vaccine against PHEV. The small interfering RNA (siRNA) technique has been suggested to have the potential to partially block the replication of PHEV by targeting the spike glycoprotein and replicase polyprotein genes (Lan et al., 2012) or cell host-specific proteins (Hu et al., 2020). Therefore, surveillance of this virus is the key to preventing the occurrence of an outbreak. In October 2020, the complete genome sequence of the virus PHEV/67 N/US/1970 collected in the United States in 1970 was determined. It represents the earliest full-length genome sequence information so far. In this study, we used this virus as a prototype to compare and analyze the full-length sequences of all other viral isolates and discussed the genome structure, genome similarity, and B cell epitopes to explore the evolutionary relationship and heredity of PHEV diversity, providing critical information for formulating disease control strategies suitable for PHEV.

## Materials and Methods

### Phylogenetic Tree Construction and Genomic Similarity Analysis

Fourteen full-length genome sequences of PHEVs isolated since 1970 were retrieved from NCBI GenBank. The viruses in this report were identified by their GenBank ID, name, country, and year of collection in a format as [GenBank ID: virus name (country-year of collection)]. The sequence of the virus PHEV/67 N/US/1970 (GenBank ID: MW165134.1) collected in 1970 was included in this analysis as a prototype reference. The phylogenetic trees were constructed using the neighbor-joining method embedded in the MEGA-X software (Kumar et al., 2004, 2018) based on PHEV full-length genomic sequence or the full-length sequence of individual ORFs. 1,000 repeated bootstrap analysis was applied to determine the percentage reliability value of each internal node of the phylogenetic tree. SimPlot ver.3.5.1 (Lole et al., 1999) was used to generate a similarity map of full-length PHEV genome.

### Prediction of Linear B Cell Epitopes in S Glycoprotein of PHEV

To analyze the potential linear B cell epitopes in PHEV S glycoprotein, we used the BepiPred-2.0 server, running under IEDB (the immune epitope database, https://www.iedb.org/; Jespersen et al., 2017). The epitope and non-epitope amino acids were determined from the crystal structure using a Random Forest algorithm (Jespersen et al., 2017). Residues with scores higher than the threshold (set to 0.5) were predicted to be part of an epitope.

### Three-Dimensional Modeling of PHEV S Glycoprotein Domains

I-TASER (Iterative Threading ASSEmbly Refinement) is a hierarchical method for protein structure modeling and structure-based functional annotation (Roy et al., 2010; Yang et al., 2015). I-TASER first identified structural templates from the RCSB protein data bank (PDB) by multiple threading approach LOMETS, then the construction of the full-length atomic models by iterative template-based fragment assembly simulations. I-TASER was performed to visualize the tertiary structure of three regions of PHEV S glycoprotein, including the NTD-containing region (aa 16–310), S1-RBD-containing region (aa 311–783), and S2 domain (aa 784–1,276).

## Results

### Phylogenetic Analysis Reveals Two Separate Evolutionary Clades of PHEVs

**Figure 1:**
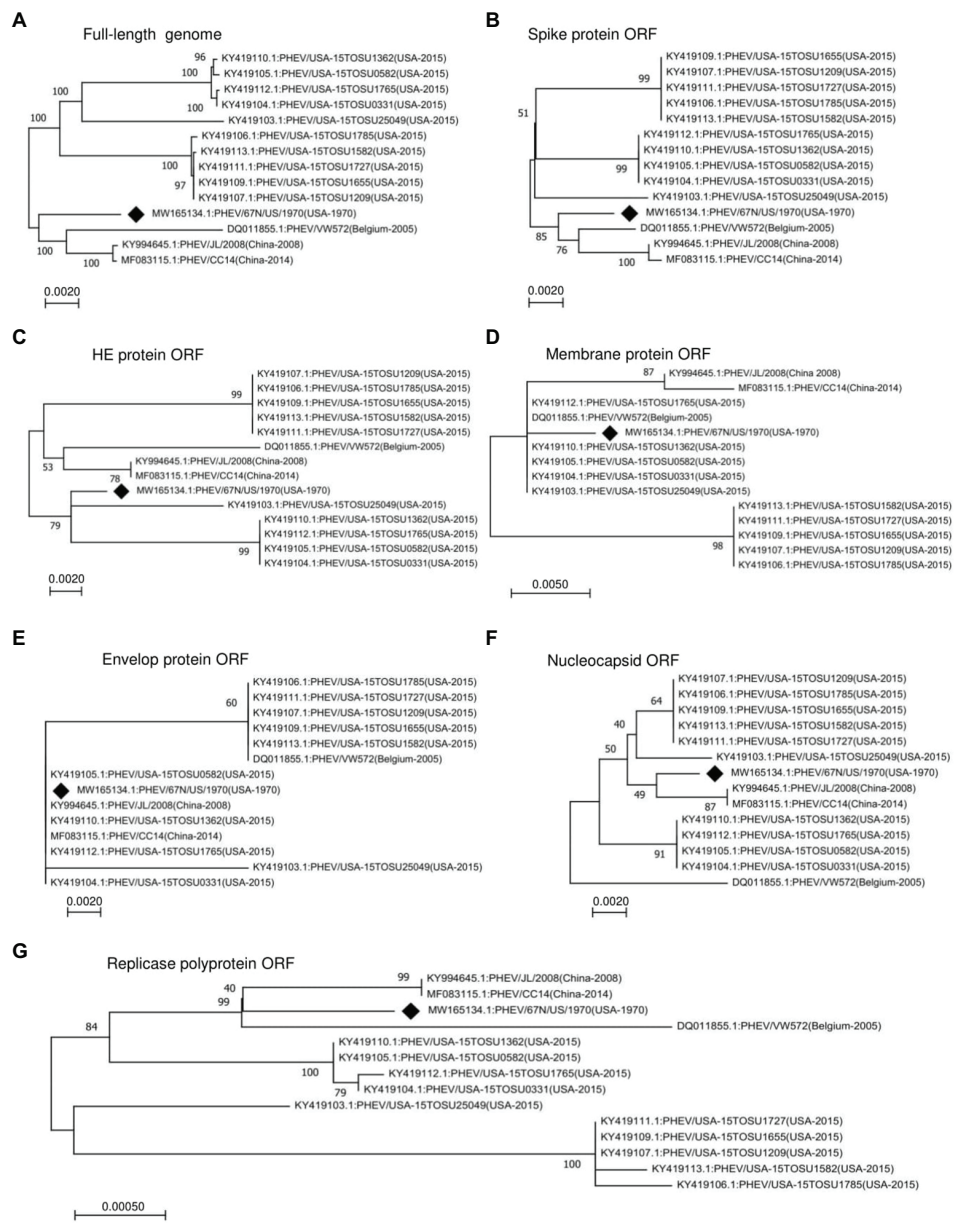
Phylogenetic analysis of 14 PHEV strains isolated between 1970 and 2015. Phylogenetic trees were constructed using the neighbor-joining algorithm in the MEGA-X software and based on **(A)** Full-length genome, or individual ORFs including **(B)** spike glycoprotein ORF, **(C)** HE glycoprotein ORF, **(D)** membrane protein ORF, **(E)** envelope protein ORF, **(F)** nucleocapsid protein ORF, and **(G)** replicase polyprotein ORF. The numbers on each branch are the bootstrap values (%) in 1000 replicates. The scale bar indicates the amino acid substitution at each position. The strain PHEV/67 N/US/1970 isolated in the United States in 1970 was labeled with a black diamond.

To determine the genomic characters, variation, and relationship between PHEV strains, we performed a phylogenetic analysis of the entire genomes or individual ORF sequences of PHEV isolates available since 1970 in the NCBI GenBank database using MEGA-X software (Kumar et al., 2004, 2018). We built phylogenetic trees based on 14 variants, isolated from the United States (11), China (2), and Belgium (1). The genomic sequence of PHEV/67 N/US/1970 virus (GenBank ID: MW165134.1) collected in the United States in 1970 was available in the NCBI GenBank database in October 2020, providing the earliest genetic information of PHEV. Therefore, this strain was included as a reference to explore the genetic diversity and evolution of PHEV over time. As indicated in Figure 1A, the phylogenetic tree of PHEV complete genome exhibits two main clades, where PHEV/67 N/US/1970 strain clustered together in one clade with strains isolated from Belgium in 2005 (PHEV/VW572, GenBank ID: DQ011855.1) and China in 2008 (PHEV/JL/2008, GenBank ID: KY994645.1) and 2014 (PHEV/CC14, GenBank ID: MF083115.1). The remaining strains isolated from the United States in 2015 (a total of 10) clustered together into another independent clade, which can be further divided into three lineage groups. To further understand the evolution of PHEV, we proceeded to individual ORF sequence-based phylogenetic analysis. In the phylogenetic tree of S glycoprotein ORF (Figure 1B), PHEV strains were sorted into two different evolutionary clades, similar to that of full-length viral genome (Figure 1A). In replicase polyprotein ORF-based phylogenetic tree, PHEV/67 N/US/1970 is still genetically closer to the strains isolated in China and Belgium (Figure 1G). However, in the phylogenetic trees based on ORFs of HE protein (Figure 1C), membrane protein (Figure 1D), envelop protein (Figure 1E), and nucleocapsid protein (Figure 1F), PHEV strains fell in different lineage groups other than groups indicated in the full-length genome phylogenetic tree (Figure 1A).

### Two Highly Variable Regions of PHEV Genome: NS2A ORF and S Glycoprotein ORF

**Figure 2:**
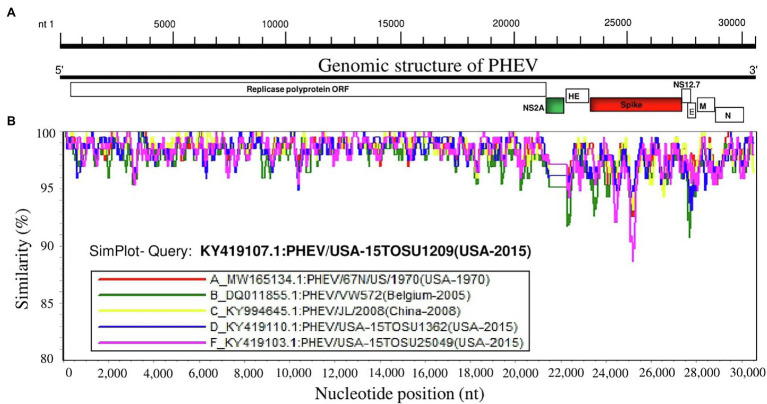
Similarity map of the full-length genome sequence of representative PHEV isolates. **(A)** Schematic diagram of the complete PHEV genomic structure. From the 5′end to the 3′ end are Replicase polyprotein ORF, accessory protein NS2A ORF, HE protein ORF, spike glycoprotein ORF, accessory protein NS12.7 ORF, envelope protein ORF (E), membrane protein ORF (M) and nucleocapsid protein ORF (N). **(B)** SimPlot similarity analysis results using SimPlot ver.3.5.1 (Lole et al., 1999). Virus PHEV/USA-15TOSU1209 (GenBank ID: KY419107.1) was used as the query sequence to compare with five other representative strains from the United States, China, and Belgium.

**Figure 3:**
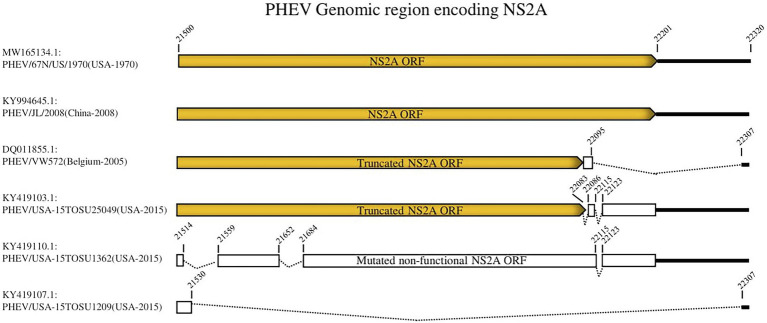
Deletion patterns in NS2A ORF region. The yellow box indicates the expression of full-length or truncated NS2A. The white box indicates the region of non-functional expression. Deleted regions are indicated with dashed lines. The numbers indicate the nucleotide positions relative to the genome of PHEV/67 N/US/1970 strain (GenBank ID: MW165134.1).

To further analyze the genetic characteristics and connectivity between PHEV strains, we conducted a genomic similarity analysis using SimPlot ver.3.5.1 software (Lole et al., 1999). Six viruses were included as representatives for each lineage group according to the whole genomic sequence-based phylogenetic tree (Figure 1A) and PHEV/USA-15TOSU1209 (GenBank ID: KY419107.1) was used as the query strain. As shown in Figure 2, the complete genome of PHEV/USA-15TOSU1209 (GenBank ID: KY419107.1) showed a great genomic similarity (>96%) with the included strains except for the region containing S glycoprotein and NS2A accessory protein ORFs that have the lowest genomic similarity, indicating their hypervariability. Sequence alignment revealed multiple deletion patterns in NS2A ORF region (Figure 3) of PHEVs isolated in the United States in 2015 and Belgium in 2005. Compared with PHEV/67 N/US/1970 (Figure 3), viruses isolated from China in 2008 (PHEV/JL/2008) and 2014 (PHEV/CC14, Data not shown) have no deletion mutations in the NS2A ORF region. In contrast, NS2A of viruses PHEV/VW572 from Belgium in 2005 and PHEV/USA-15TOSU25049 from the United States in 2015 has small deletions of 39 and 38 amino acids at the C-terminus, respectively. The representative viruses isolated in the United States in 2015 (PHEV/USA-15TOSU1362 and PHEV/USA-15TOSU1209) exhibited multiple deletions all through the NS2A ORF region, resulting in a non-functional expression of NS2A (Figure 3).

### Linear B Cell Epitopes in S Glycoprotein of PHEV

**Figure 4:**
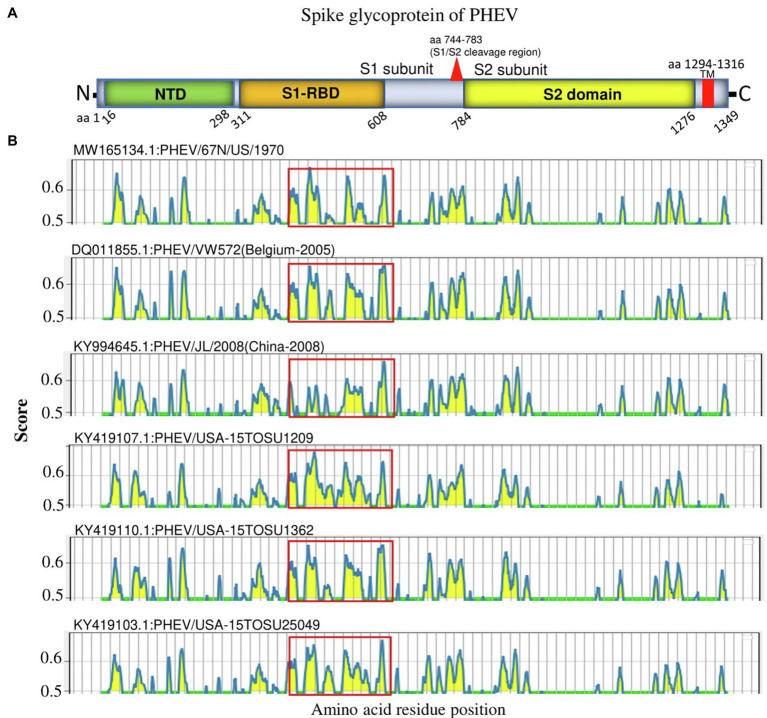
Linear B cell epitope map of the full-length S glycoprotein of PHEV. **(A)** Diagram of the main features of PHEV S glycoprotein, including NTD (aa 16–298), S1-RBD (aa 311–608), S2 domain (aa 784–1,276), and transmembrane domain (TM; aa 1,294–1,316). Numbers refer to the amino acid positions corresponding to PHEV/67 N/US/1970 S glycoprotein. **(B)** Mapping of linear B cell epitopes using the BepiPred-2.0 server. The *Y*-axes represents the residue score, and the X-axes represents the amino acid position, which is also relative to the diagram on the top **(A)**. The predicted epitopes are indicated in yellow.

**Figure 5:**
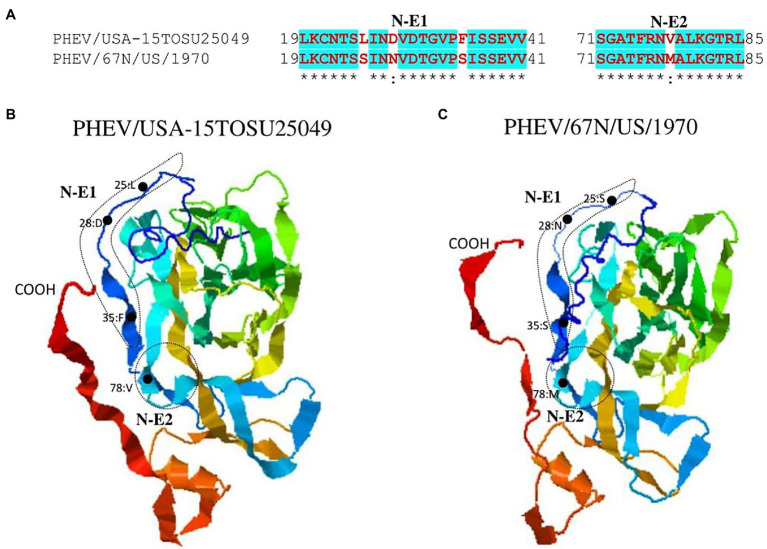
Comparison of linear B cell epitopes affected by amino acid variations in the NTD of S glycoprotein between PHEV/USA-15TOSU25049 and PHEV/67 N/US/1970. **(A)** Amino acids of the predicted linear B cell epitope are indicated with bold red letters, while numbers refer to their positions relative to the corresponding full-length S glycoprotein. Three-dimensional view of epitopes of PHEV/USA-15TOSU25049 **(B)** and PHEV/67 N/US/1970 **(C)**. The tertiary structure was modeled using I-TASER. The predicted epitopes are circled, and amino acid variations are marked with black dots.

**Figure 6:**
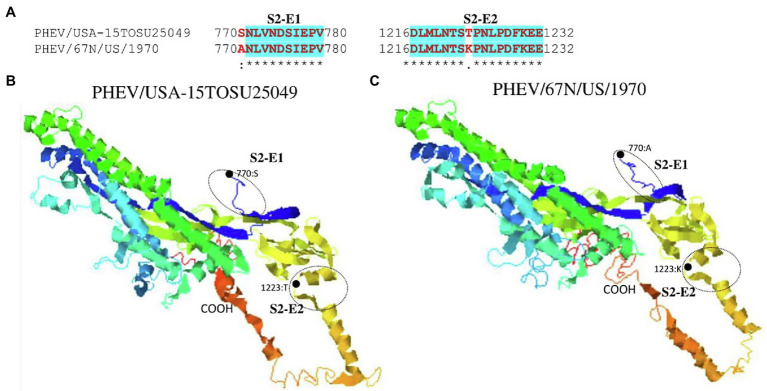
Comparison of linear B cell epitopes affected by amino acid variations in the S2 domain between PHEV/USA-15TOSU25049 and PHEV/67 N/US/1970. **(A)** Amino acids of the predicted linear B cell epitope are indicated with bold red letters, while numbers refer to their positions relative to the full-length S glycoprotein. Three-dimensional view of epitopes in the S2 domain of PHEV/USA-15TOSU25049 **(B)** and PHEV/67 N/US/1970 **(C)**. The predicted epitopes are circled, and the amino acid variations are marked with black dots.

**Figure 7:**
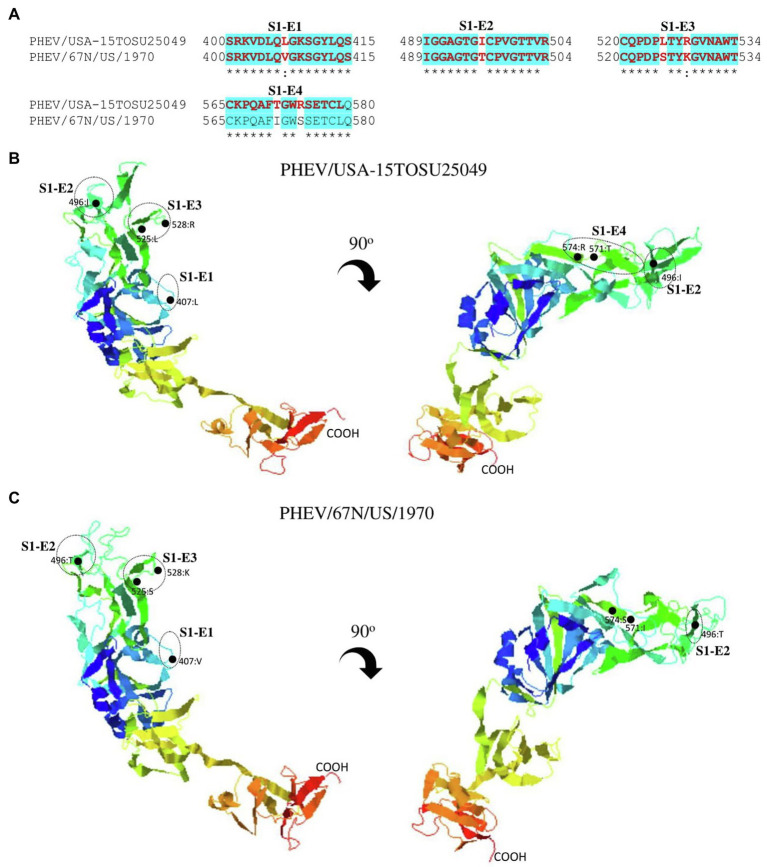
Comparison of linear B cell epitopes affected by amino acid variations in the S1-RBD between PHEV/USA-15TOSU25049 and PHEV/67 N/US/1970. **(A)** Amino acids of the predicted linear B cell epitope are indicated with bold red letters, while numbers refer to their positions relative to the corresponding full-length S glycoprotein. Three-dimensional view of epitopes in the S1-RBD of PHEV/USA-15TOSU25049 **(B)** or PHEV/67 N/US/1970 **(C)**. The predicted epitopes are circled, and amino acid variations are marked with black dots.

S glycoprotein of coronaviruses is the major viral antigen that induces the generation of neutralizing antibodies during host infection, making it a major target protein for vaccine development (Du et al., 2009). Since S glycoprotein ORF of PHEV is found relatively highly variable (Figure 2), we assessed the effects of detected genomic variations on the antigenicity of S glycoprotein. For this aim, the linear B-cell epitopes on S glycoprotein of six representative viruses analyzed in genomic similarity analysis (Figure 2) were predicted using BepiPred-2.0 epitope prediction server (Jespersen et al., 2017). As indicated in Figure 4, a significant difference in the distribution of potential B cell epitopes was seen in S1-RBD (aa 311–608), whereas there were minor differences for the remaining part of S glycoprotein. Viruses PHEV/USA-15TOSU25049 (GenBank ID: KY419103.1) and PHEV/67 N/US/1970 (GenBank ID: MW165134.1) isolated in the same country (United States), were sorted into separate evolutionary clades in either full-length genome-based (Figure 1A) or S glycoprotein ORF-based phylogenetic tree (Figure 1B). However, compared with other viral strains, both viruses were genetically closer to each other in the S glycoprotein ORF-based phylogenetic tree (Figure 1B). Thus, the three-dimensional structures of NTD, S1-RBD, and S2 domains of these two viruses were modeled using I-TASER (Roy et al., 2010; Yang et al., 2015) to visualize the minimal amino acid variations seen in two main evolutionary clades in the potential linear B cell epitopes. For NTD or S2 domains of viruses PHEV/USA-15TOSU25049 and PHEV/67 N/US/1970, only two linear B cell epitopes were affected by amino acid variations (Figures 5 and 6, respectively), whereas four predicted epitopes were affected in S1-RBD (Figure 7). The mutations T571I and R574S eliminated the epitope S1-E4 in PHEV/67 N/US/1970. No amino acid insertions or deletions were seen in the potential linear B cell epitopes. In summary, the S1-RBD may be one of the main factors driving the antigenic drift of S glycoprotein of PHEV.

## Discussion

Although PHEV was one of the earliest identified and isolated swine CoVs, it remains after decades poorly documented and less studied with limited availability of genome sequences. In this report, we conducted a phylogenetic analysis based on the complete genome of the earliest isolated PHEV strain in the United States in 1970, available in October 2020, with 13 other isolates from different countries, including 10 from the United States, two from China, and one from Belgium. The phylogenetic tree showed two main clades, where the earliest United States strain (PHEV/67 N/US/1970) clustered together with the two strains from China and one from Belgium, whereas the other 10 United States variants (isolated in 2015) clustered together in another independent clade (Figures 1A,B), demonstrating the existence of two different circulating PHEV groups in the United States. Furthermore, NS2A and S glycoprotein ORFs were highly variable in PHEV genome, altering the expression of a normal NS2A and the composition of linear B cell epitopes in the S glycoprotein, particularly S1-RBD.

Compared with other *Beta-CoVs* from a supposed common ancestor such as human coronavirus OC43 and bovine coronavirus (Vijgen et al., 2006), NS2A of PHEV is shorter with a missing of ~84 amino acids at the C-terminus (Vijgen et al., 2006; Lau et al., 2012). NS2A belongs to the 2H phosphoesterase superfamily (Mazumder et al., 2002; Snijder et al., 2003), but its function in coronavirus replication is still unknown. In previous reports, mutation deletions in NS2A of PHEVs have been linked to severe respiratory manifestations in pigs (Lorbach et al., 2017; Wang et al., 2019), suggesting that the detected NS2A deletions might be involved in the pathogenicity of PHEV-mediated respiratory syndrome.

Besides NS2A, the S glycoprotein of coronaviruses is a direct target of cellular and humoral immune responses and a vaccine design focus since it represents the main viral surface antigen (Buchholz et al., 2004; Zhi et al., 2005; Tortorici et al., 2019; Dai and Gao, 2021). S glycoprotein mutations help escape the immune response and promote animal-human transmission through adaptation potentials (Zhang et al., 2006). The hypervariability of S glycoprotein is the main cause of changes in tissue tropism (Gallagher and Buchmeier, 2001; Hulswit et al., 2016). The deletion of about 216 or 227 amino acid residues in the N-terminus of S glycoprotein of TGEV has causally changed the enteric tissue tropism to a respiratory tissue tropism (named PRCV; Laude et al., 1993; Vaughn et al., 1995), which may be linked to the distribution of receptors over tissues or their different usage by different CoV strains (Whitworth et al., 2019). PDEV with the large deletion in the N terminus of S glycoprotein (about 194–216 amino acid residues) has shown a partial attenuation, although the tissue tropism was not altered (Hou et al., 2017). During PHEV infection, a crucial interaction between S glycoprotein and NCAM was identified (Gao et al., 2010), with a fragment (aa 291–548) in S1 subunit as a minimum number of amino acids essential to binding NCAM (Dong et al., 2015). Contrary to NS2A ORF region of PHEV, which showed significant deletions (Figure 3), S glycoprotein ORF did not undergo any deletion pattern.

Porcine hemagglutinating encephalomyelitis virus is the unique porcine neurotropic CoV with the potential to invade the central nervous system. Neurologic symptoms in PHEV-infected piglets with respiratory or digestive signs indicate the involvement of neurobiological pathways, which might be related to multiple cellular receptors in tissues of three major above mentioned systems. In the latest report, cell-surface glycans, including sialic acid (SA) and heparan sulfate (HS) were found to act as critical cellular factors involved in the attachment of PHEV (Hu et al., 2022). Altogether, this indicates the complexity of the underlying mechanisms of PHEV S glycoprotein interaction with cellular receptors.

S glycoprotein is a great inducer of neutralizing antibodies and encompasses multiple epitopes, making it an important target and informative source for CoVs vaccine design (Du et al., 2009), including the licensed vaccines against SARS-CoV-1 (Du et al., 2009) and the SARS-CoV-2 vaccines used to fight against the ongoing COVID-19 pandemic (Samrat et al., 2020; Dai and Gao, 2021). Like multiple coronaviruses, the hypervariability and the high dissimilarity of PHEV S glycoprotein ORF detected in this study impacted the potential B cell epitopes. The mutations affected at least eight potential linear B cell epitopes on S glycoprotein, half of which were located in the S1-RBD. This information is of great value for the vaccine design and the surveillance of genetic diversity of PHEV.

## Data Availability Statement

Publicly available datasets were analyzed in this study. This data can be found here: Porcine hemagglutinating encephalomyelitis virus strain PHEV/67 N/US/1970, complete genome GenBank: MW165134.1.

## Author Contributions

AB, YG, and LX: conceptualization. AB, YG, PW, YL, and RS: data analysis. AB, YG, and LX: visualization and writing. CW and LX: administration. AB, YG, and LX: manuscript revision. All authors contributed to the article and approved the submitted version.

## Conflict of Interest

The authors declare no conflict of interest concerning the research, authorship, or publication of the article.

## Publisher’s Note

All claims expressed in this article are solely those of the authors and do not necessarily represent those of their affiliated organizations, or those of the publisher, the editors and the reviewers. Any product that may be evaluated in this article, or claim that may be made by its manufacturer, is not guaranteed or endorsed by the publisher.
